# Association between the prognosis and comorbidity of active cancer in chronic thromboembolic pulmonary hypertension

**DOI:** 10.1186/s12890-024-03460-5

**Published:** 2025-01-02

**Authors:** Hiroyuki Fujii, Yu Taniguchi, Yuichi Tamura, Miki Sakamoto, Sachiyo Yoneda, Kenichi Yanaka, Noriaki Emoto, Ken-ichi Hirata, Hiromasa Otake

**Affiliations:** 1 Division of Cardiovascular Medicine, Department of Internal Medicine, Kobe University Graduate School of Medicine, 7-5-2 Kusunoki-cho, Chuo-ku, Kobe, 6500017 Japan; 2 Pulmonary Hypertension Center, International University of Health and Welfare Mita Hospital, Tokyo, Japan; 3 Laboratory of Clinical Pharmaceutical Science, Kobe Pharmaceutical University, Kobe, Japan

**Keywords:** Chronic thromboembolic pulmonary hypertension, Cancer, Prognosis, Balloon pulmonary angioplasty

## Abstract

**Background:**

Prognosis of chronic thromboembolic pulmonary hypertension (CTEPH) has improved after the availability of balloon pulmonary angioplasty (BPA) and approved drugs. However, the clinical effects of cancer, which is one of the associated medical conditions of CTEPH, remain unclear. We aimed to investigate prognosis in patients with CTEPH and comorbid cancer.

**Methods:**

Between January 2011 and December 2022, data of 264 consecutive patients with CTEPH who were treated with pulmonary endarterectomy, BPA, or medical therapy were retrospectively analyzed. The patients were allocated, based on the comorbidity of cancer as of December 2022, into the cancer (*n* = 47) and non-cancer (*n* = 217) groups. In the cancer group, active and non-active cancers were identified in 30 (64%) and 17 (36%) patients, respectively.

**Results:**

The baseline characteristics, hemodynamics, and treatments were similar between the groups. More than half of the cancer were diagnosed within two years before or after CTEPH diagnosis. Twenty-seven patients died during the study period. Among them, 13 (48%) and 7 (26%) died of cancer and right heart failure, respectively. The 5-year survival rate was lower in the cancer group than in the non-cancer group (67.8% vs. 94.5%, *p* < 0.001). In the active cancer group, the 5-year survival rate was also lower than that in the non-active cancer and non-cancer groups (52.0% vs. 99.5%, *p* < 0.001 and 52.0% vs. 92.3%, *p* < 0.001, respectively). Multivariate Cox hazard analysis revealed that hemodialysis (*p* < 0.001) and cancer (*p* < 0.001) were independently associated with poor survival.

**Conclusion:**

Patients with CTEPH rarely die of right heart failure, even if hemodynamically severe at diagnosis in the modern management era. However, patients with CTEPH frequently have comorbid cancer, which may be a strong prognostic factor.

## Background

Chronic thromboembolic pulmonary hypertension (CTEPH) is characterized by stenosis and pulmonary artery obstruction caused by non-resolving organized thromboemboli combined with variable microvasculopathy, leading to elevated pulmonary vascular resistance, severe pulmonary hypertension (PH), and right heart failure [1–3]. In the past, when specific treatments of CTEPH were not available, prognosis in patients with CTEPH was remarkably poor, with a 5-year survival rate of 10% in patients with a mean pulmonary artery pressure of > 50 mmHg [4]. Surgical pulmonary endarterectomy (PEA) is the standard treatment for managing patients with operable CTEPH [5, 6]. Balloon pulmonary angioplasty (BPA), an endovascular procedure to widen narrowed or obstructed pulmonary arteries, and approved medical therapies have become established treatments of non-operable CTEPH in the current guidelines for PH [5]. Almost all types of CTEPH can be treated with appropriate indications for PEA, BPA, or medical therapy in the modern management era, in which several treatment options are available. Almost normal hemodynamics can be achieved after these interventional treatments. Moreover, these hemodynamic improvements translate into excellent survival of patients with operable and non-operable CTEPH. Patients with CTEPH rarely die of right heart failure [7].

In recent years, the association between CTEPH and cancer has attracted attention. The risk of thrombosis is significantly higher in patients with malignant tumors than in healthy individuals [8]. Malignancy is a risk factor for the development of thrombosis and a medical condition associated with CTEPH [9, 10]. Nakamura et al. reported that the comorbidity of cancer was associated with poor prognosis in patients with CTEPH [11].

However, the frequency of cancer-related comorbidities and their prognostic relevance in patients with CTEPH have rarely been reported. We aimed to evaluate the comorbidity of cancer and its clinical effects on prognosis in patients with CTEPH in the modern management era.

## Methods

This retrospective study was conducted in compliance with the principles of the Declaration of Helsinki. The study protocol was approved by the Ethics Committee of Kobe University Hospital (approval number: B230114). All enrolled patients were provided with the option to opt out if they did not wish to participate. The requirement for written informed consent was waived because the data were retrospectively collected. Data supporting the findings of this study are available from the corresponding author upon request.

### Patients/study design

This retrospective observational study was conducted in consecutive patients with CTEPH who were diagnosed, treated, and followed up in Kobe University Hospital (Kobe, Japan) from January 2011 (commencement of our BPA program) to December 2022. All patients were diagnosed with CTEPH according to the established clinical guidelines [1, 12]. Diagnosis was made based on medical history, physical examination, ventilation-perfusion lung scan, multidetector computed tomographic pulmonary angiography, right heart catheterization (RHC), and selective pulmonary angiography. Clinical assessments, including hemodynamic characteristics assessed using RHC, arterial blood gas analysis, functional status based on the New York Heart Association functional class (NYHA-FC), and exercise capacity using the 6-min walk test (6-MWT), were performed at the time of CTEPH diagnosis. Patients who underwent PEA or BPA were re-evaluated by RHC three months after the last session. Vital status was assessed at the last follow-up visit. In patients without any follow-up for > 3 months, the mortality status was determined by making a contact via telephone. The primary objective of this study was to investigate the clinical effects of comorbid cancer on prognosis in patients with CTEPH.

### Treatment of chronic thromboembolic pulmonary hypertension

In November 2001, we initiated surgical PEA in patients with operable CTEPH. Non-operated patients were treated with oral anticoagulants alone or with pulmonary arterial hypertension (PAH) drugs, according to their clinical status and treatment availability. In March 2011, we launched a BPA program for managing patients with non-operable CTEPH. Patients diagnosed since 2011 could undergo PEA or BPA at an early stage after diagnosis. To avoid bias regarding the effects of PEA or BPA on incident patients, patients diagnosed since 2011, when all treatments were available, were enrolled in this study.

The assessment of treatment strategies, including PEA, BPA, medical therapy, and a combination of these, was performed by a multidisciplinary team of experts, including experienced BPA interventionists and PEA surgeons, as recommended by the clinical guidelines for PH that were current during the observational period [5, 12, 13]. PEA was performed for surgically accessible lesions; BPA was performed for non-operable lesions. Patients who refused invasive treatments and those with: extremely old age (> 90 years), significantly severe comorbidities, and malignancy whose expected prognosis was < 6 months were not offered interventional treatment with PEA or BPA.

### History, status, and definition of comorbid cancer

Patients with malignant cancer were defined as those with a history of cancer as of December 2022. In addition to the baseline clinical, functional, and hemodynamic characteristics of CTEPH, we retrospectively recorded the cancer type and stage, treatment details (i.e., surgery, radiation, chemotherapy, and hormone therapy), and timing of cancer diagnosis. We defined active cancer as: cancer diagnosed within the previous six months; recurrent, regionally advanced, or metastatic cancer; cancer for which treatment had been administered within six months; or hematological cancer that was not in complete remission [14]. We classified cancer into groups as active cancer and non-active cancer based on this definition.

### Statistical analysis

All statistical analyses were performed using the Statistical Package for Social Sciences version 26.0 (IBM, Armonk, NY, USA) and GraphPad Prism version 5 (GraphPad Software, La Jolla, CA, USA). Continuous variables are expressed as means ± standard deviations. Differences in continuous variables, such as age, 6-MWT distance, hemodynamic data, and oxygenation parameters, were compared using the paired Student’s t-test for normally distributed variables and the Mann–Whitney U test for non-normally distributed variables. Categorical variables, such as sex, NYHA-FC, and treatments, are expressed as numbers and percentages and were compared using the χ^2^ test for independence or using Fisher’s exact test when the expected counts were < 5. Regarding survival analysis, the date of CTEPH diagnosis was used as the starting point to determine the length of survival. The cutoff date was December 31, 2022. The Kaplan–Meier method was used to estimate the overall survival at each interval. Univariate analysis based on the Cox proportional hazards model was used to examine the effect of each variable (baseline clinical and hemodynamic characteristics, management, and cancer status) on survival. Variables with p values < 0.2 from the univariate analysis were fitted into a multivariate model to examine the independent effect of each variable on survival. In these Cox hazard analyses, comorbid cancer was assigned as a time-varying covariate because of the time lapse between the diagnosis of CTEPH and that of cancer, which might have biased the effects of comorbid cancer on survival. Considering all analyses, the level of statistical significance was set at *p* < 0.05.

## Results

### Patient population

**Fig. 1:**
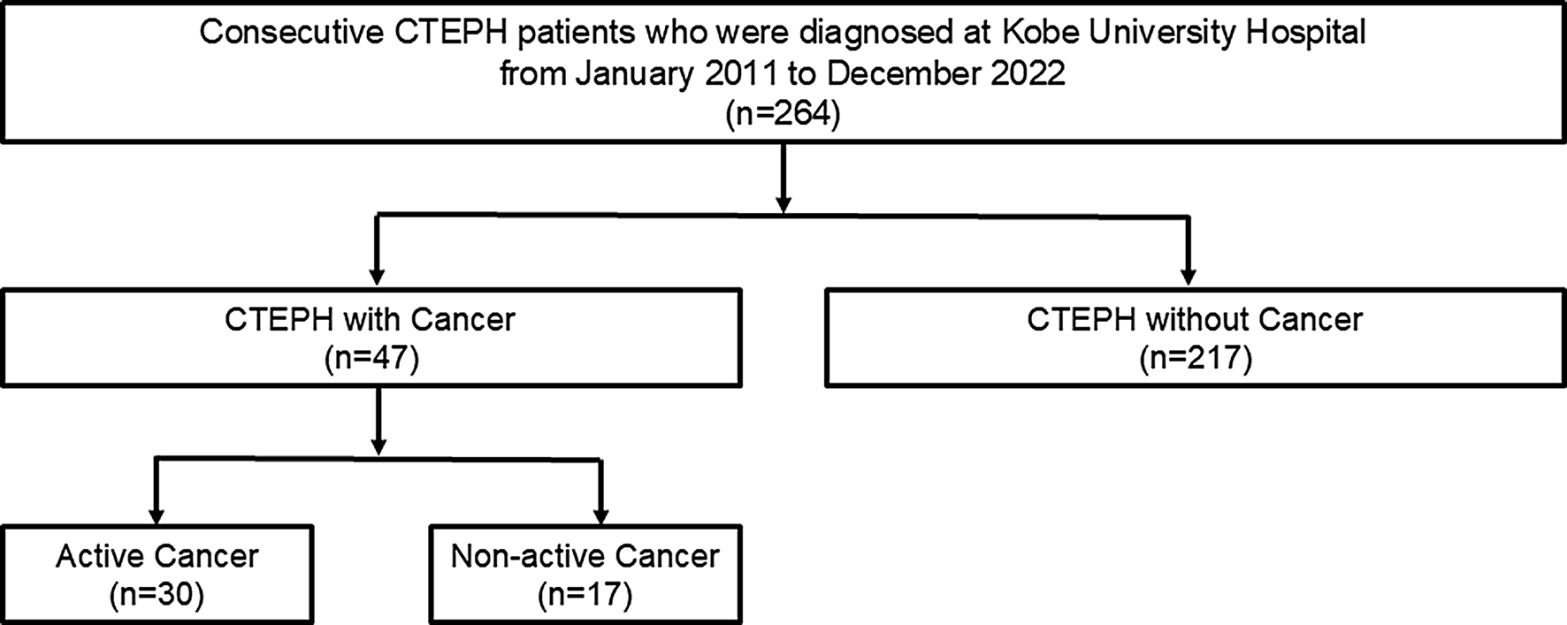
Patient study cohort. CTEPH, chronic thromboembolic pulmonary hypertension

Between January 2011 and December 2022, 264 consecutive patients diagnosed with CTEPH at Kobe University Hospital were enrolled in this study. Of these, 65 (25%) underwent PEA, and 169 (64%) underwent BPA. Among the operated patients, 32 (12%) underwent only PEA and the remaining 33 (13%) underwent additional BPA due to residual PH or symptoms after PEA. Thirty (11%) patients were treated with medication only because of the patients’ refusal of interventional therapy and extremely advanced age or severe comorbidities. Of the 264 patients included in the analysis, 47 (18%) were classified into the cancer group and 217 (82%) into the non-cancer group based on the history of cancer as of December 2022. In the cancer group, 30 patients (64%) were identified with active cancer and 17 (36%) with non-active cancer. The patient cohort is shown in Fig. 1. The baseline characteristics of the patients in the cancer and non-cancer groups are summarized in Table 1. The baseline characteristics and treatments of PH were similar between the groups. Hemodynamics and exercise capacities are presented in Table 2. Hemodynamics and exercise capacity in both groups were similar. The mean pulmonary arterial pressure in patients treated with PEA or BPA decreased significantly in both groups (cancer group: 38.7 ± 10.4 mm Hg to 19.2 ± 4.4 mm Hg; *p* < 0.001 and non-cancer group: 37.3 ± 10.9 mm Hg to 19.9 ± 5.6 mm Hg; *p* < 0.001). There was no difference in the treatment effect of BPA or PEA between the cancer and non-cancer groups. During the study period, more patients died in the cancer group compared with those in the non-cancer group (28% vs. 6%, respectively; *p* < 0.001).

**Table 1:** Baseline characteristics of the patient population

Variable	Overall population (*n* = 264)	Cancer group (*n* = 47)	Non-cancer group (*n* = 217)	*p* value*
*Baseline characteristics*				
Age, y	67 ± 13	67 ± 12	67 ± 13	0.9555
Men (n, %)	67 (25%)	13 (28%)	54 (25%)	0.6932
BMI, kg/m^2^	23.1 ± 3.8	23.6 ± 3.3	23.0 ± 3.9	0.2980
NYHA FC (I,II/III,IV)(%)	27/73	31/69	28/72	0.7263
Previous DVT (n, %)	43 (16%)	11 (23%)	32 (15%)	0.1461
Previous acute PE (n, %)	77 (29%)	18 (38%)	59 (27%)	0.1297
Smoking (n, %)	66 (25%)	14 (30%)	52 (24%)	0.4051
*Comorbidities*				
Hypertension (n, %)	77 (29%)	9 (19%)	68 (31%)	0.0963
Diabetes (n, %)	33 (13%)	7 (15%)	26 (12%)	0.5859
Atrial fibrillation (n, %)	15 (6%)	2 (4%)	13 (6%)	0.6428
Coronary artery disease (n, %)	7 (3%)	3 (6%)	4 (2%)	0.0796
Dyslipidemia (n, %)	65 (25%)	11 (23%)	54 (25%)	0.8316
Hemodialysis (n, %)	6 (2%)	2 (4%)	4 (2%)	0.3163
*Treatment*				
PEA (n, %)	65 (25%)	9 (19%)	56 (26%)	0.3387
BPA (n, %)	202 (76%)	38 (81%)	164 (76%)	0.4412
PEA + BPA (n, %)	33 (13%)	4 (9%)	29 (13%)	0.3636
Only medication (n, %)	30 (11%)	4 (9%)	26 (12%)	0.4985
HOT, n (%)	101(38%)	21 (45%)	80 (37%)	0.3195
*Medical treatment*				
Warfarin (n, %)	200 (76%)	33 (70%)	167 (77%)	0.3297
DOAC (n, %)	64 (22%)	14 (30%)	50 (23%)	0.3297
sGC Stimulator (n, %)	125 (47%)	26 (55%)	99 (46%)	0.3947
ERA (n, %)	37 (14%)	3 (6%)	34 (16%)	0.2043
PDE5-i (n, %)	22 (8%)	5 (11%)	17 (8%)	0.8415
Prostacyclin analog (n, %)	31 (12%)	4 (9%)	27 (8%)	0.3943

*List of abbreviations*: BMI: body mass index; NYHA FC: New York Heart Association functional class; DVT: deep venous thrombosis; PE: pulmonary embolism; PEA: pulmonary endarterectomy; BPA: balloon pulmonary angioplasty; HOT: home oxygen therapy; DOAC: direct oral anticoagulant; sGC: soluble guanylate cyclase; ERA: endothelin-receptor antagonists; PDE5-i: phosphodiesterase type-5 inhibitors

Data are given as mean ± standard deviation

* Comparison between a Cancer group and a non-Cancer group

**Table 2:** Baseline exercise capacities and hemodynamics of the patient population

Variable	Overall population (*n* = 264)	Cancer group (*n* = 47)	Non-cancer group (*n* = 217)	*p* value*
*Exercise capacities*				
6MWD (m)	325.7 ± 112.6	341.2 ± 95.2	322.5 ± 115.8	0.3468
Baseline SpO_2_ (%)	94.2 ± 3.0	94.4 ± 3.1	94.2 ± 3.0	0.7876
Minimum SpO_2_ (%)	86.3 ± 5.3	86.5 ± 5.1	86.2 ± 5.4	0.7510
*Hemodynamics*				
Mean RAP, mmHg	5.1 ± 3.8	5.6 ± 3.9	5.0 ± 3.8	0.3778
Systolic PAP, mmHg	65.8 ± 19.6	68.0 ± 19.5	65.3 ± 19.6	0.4109
Diastolic PAP, mmHg	21.7 ± 7.8	22.3 ± 7.3	21.6 ± 7.9	0.6081
Mean PAP, mmHg	37.5 ± 10.8	38.7 ± 10.4	37.3 ± 10.9	0.4504
PAWP, mmHg	8.2 ± 3.8	8.0 ± 2.9	8.2 ± 4.0	0.7114
Cardiac Index, L/min/m^2^	2.5 ± 0.7	2.5 ± 0.8	2.5 ± 0.7	0.6021
PVR, dyne/sec/cm^− 5^	747.2 ± 438.1	727.3 ± 362.4	751.3 ± 452.5	0.7444
SaO_2_ (%)	90.7 ± 4.9	90.5 ± 4.9	90.8 ± 4.9	0.7005
SvO_2_ (%)	63.5 ± 8.8	62.8 ± 11.1	63.6 ± 8.2	0.5695
PaO_2_, mmHg	61.0 ± 13.1	59.8 ± 13.6	61.3 ± 13.1	0.5237
PaCO_2_, mmHg	36.8 ± 5.1	35.9 ± 4.5	37.0 ± 5.3	0.2350
*Death (n*,* %)*	27 (10%)	13 (28%)	14 (6%)	< 0.0001

*List of abbreviations*: 6MWD: 6-minute walk distance; SpO_2_: percutaneous oxygen saturation; RAP: right atrial pressure; PAP: pulmonary artery pressure; PAWP: pulmonary artery wedge pressure; PVR: pulmonary vascular resistance; SaO_2_: arterial oxygen saturation; SvO_2_: mixed venous oxygen saturation; PaO_2_: partial pressure of arterial oxygen; PaCO_2_: partial pressure of arterial carbon dioxide

Data are given as mean ± standard deviation

* Comparison between a Cancer group and a non-Cancer group

### Cancer information

**Fig. 2:**
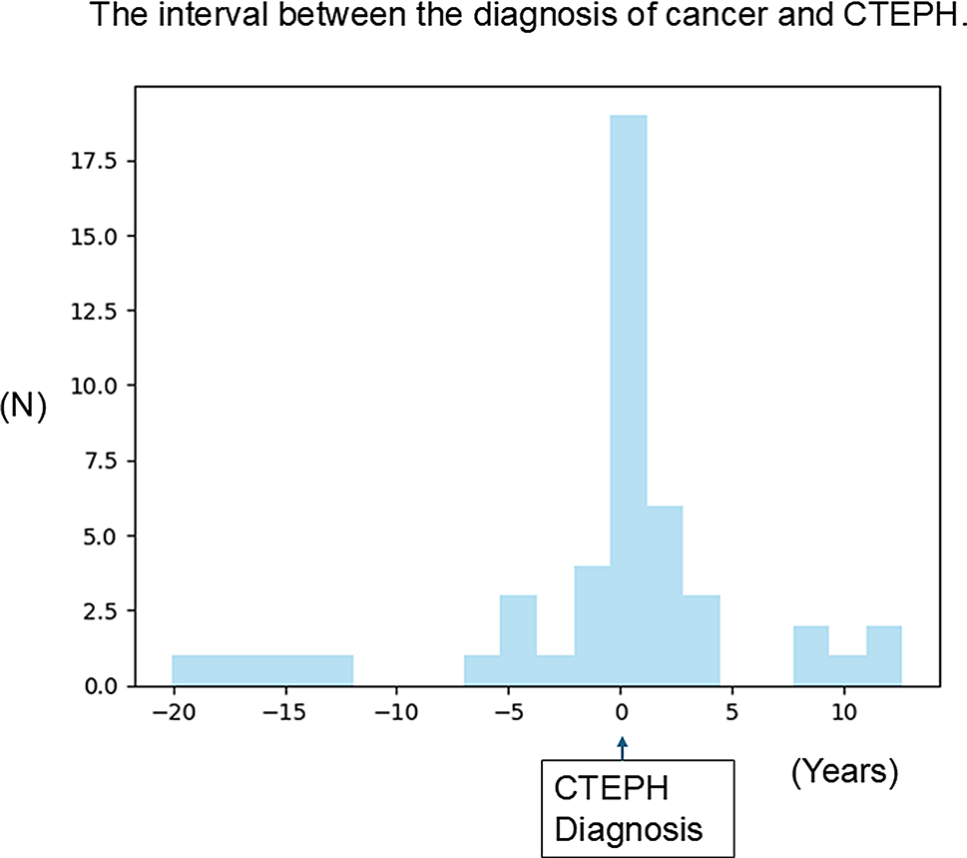
Distribution of time interval between the diagnosis of CTEPH and cancer. CTEPH, chronic thromboembolic pulmonary hypertension

Detailed cancer information (e.g., cancer type, stage, and treatment) in the cancer group is summarized in Table 3. A history of cancer was observed in 18% of the patients with CTEPH, mainly lung, hematologic malignancy, breast, and colon cancers. Regarding the timing of malignant cancer diagnosis, 23% of the patients were diagnosed with cancer before CTEPH diagnosis, 55% were diagnosed with cancer at almost the same time (within approximately 2 years) of CTEPH diagnosis, and 21% were diagnosed with cancer after CTEPH diagnosis. A graph of the interval between the diagnosis of cancer and CTEPH is shown in Fig. 2.

**Table 3:** Information of cancer

	Cancer group (*n* = 47)
*Cancer type*	
Bile duct	1 (2.1%)
Brain	2 (4.3%)
Breast	7 (14.9%)
Colon	6 (12.8%)
Esophageal	1 (2.1%)
Gastric	1 (2.1%)
Hematological malignancy	8 (17.0%)
Liver	1 (2.1%)
Lung	11 (23.4%)
Pancreas	2 (4.3%)
Prostate	2 (4.3%)
Rectum	1 (2.1%)
Renal	1 (2.1%)
Thyroid	1 (2.1%)
Uterine	4 (8.5%)
Others	1 (2.1%)
*Cancer stage*	
I	7 (14.9%)
II	6 (12.8%)
III	9 (19.1%)
IV	7 (14.9%)
Unknown	18 (38.3%)
*Metastasis*	7 (14.9%)
*Treatment*	
Surgical therapy	31 (66.0%)
Radiation therapy	5 (10.6%)
Chemotherapy	18 (38.3%)
Hormone therapy	4 (8.5%)

### Survival

**Fig. 3:**
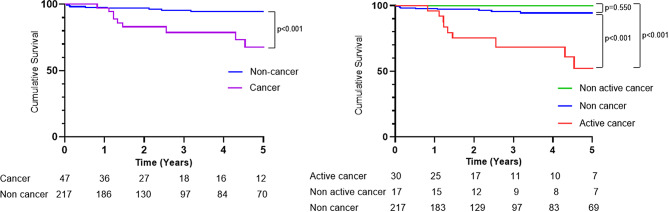
Kaplan–Meier estimates of 5-year survival in patients with CTEPH and cancer (cancer, *n* = 47) (purple line) and patients with CTEPH without cancer (non-cancer, *n* = 217) (blue line). Kaplan–Meier estimates of 5-year survival in patients with CTEPH and Active cancer (Active cancer, *n* = 30) (red line), patients with CTEPH and Non active cancer (Non active cancer, *n* = 17) (green line) and patients with CTEPH without cancer (non cancer, *n* = 217) (blue line)

During a median follow-up of 28.2 (interquartile range, 16.1 − 69.8) months, 27 of 264 (10%) patients died. Thirteen of 47(27.7%) in the cancer group and 14 of 217 the (6.5%) in non-cancer group died respectively (*p* < 0.001). Among them, 13 (48%) died of cancer and 7 (26%) died of right heart failure. The 5-year survival rate in the cancer group was lower than that in the non-cancer group (67.8% vs. 94.5%; 95% confidence interval [CI], 0.08–0.60; *p* < 0.001 using the Cox–Mantel log-rank test). The 5-year survival rate in the active cancer group was also lower than that in the non-active cancer and non-cancer groups (52.0% vs. 99.5%; 95% CI, 0.03–0.26; *p* < 0.001, 52.0% vs. 92.3%; 95% CI, 0.04–0.47; *p* < 0.001 respectively, using the Cox–Mantel log-rank test) (Fig. 3).

Table 4 summarizes the results of Cox hazard analysis of the factors associated with survival. Using univariate analysis, advanced age (hazard ratio [HR], 1.065; 95% CI, 1.02 − 1.11; *p* = 0.002), hemodialysis (HR, 4.723; 95% CI, 4.08 − 18.76; *p* < 0.001), cancer (HR, 8.749; 95% CI, 4.08 − 18.76; *p* < 0.001), and 6-MWT distance (HR, 0.995; 95% CI, 0.99 − 1.00; *p* = 0.025) were significantly associated with survival. Multivariate Cox hazards analysis revealed that hemodialysis (HR, 503.38; 95% CI, 12.97 − 19533.48; *p* < 0.001) and cancer (HR, 44.34; 95% CI, 10.17 − 193.37; *p* < 0.001) were independently associated with poor survival.

**Table 4:** Univariate and multivariable Cox hazard analysis of prognostic variables in overall CTEPH patients

	Univariate				Multivariable		
Variable	HR	95% CI	*p* value		HR	95% CI	*p* value
***Baseline characteristics***							
Age (years)	1.065	1.024–1.107	0.002				
Male	1.185	0.525–2.676	0.683				
***Comorbidities***							
Hypertension	0.376	0.113–1.256	0.112				
Diabetes	1.188	0.448–3.147	0.729				
Atrial Fibrillation	1.685	0.572–4.965	0.344				
Hemodialysis	4.723	1.405–15.870	0.012		503.383	12.972–19533.476	< 0.001
Cancer	8.749	4.081–18.757	< 0.001		44.338	10.166–193.372	< 0.001
***Baseline hemodynamics***							
Mean RAP (mmHg)	1.050	0.962–1.148	0.275				
Mean PAP (mmHg)	0.995	0.961–1.030	0.770				
Cardiac Index (L/min per m^2^)	0.826	0.468–1.458	0.510				
***Exercise capacity***							
6MWD (m)	0.995	0.991–0.999	0.025				
***Treatments***							
PEA	0.683	0.283–1.648	0.396				
BPA	0.487	0.221–1.071	0.074				
sGC stimulator	0.981	0.556–1.732	0.948				
ERA	0.892	0.542–1.468	0.653				
PDE5-i	1.003	0.566–1.777	0.992				
Prostacyclin analog	0.885	0.431–1.816	0.738				

*List of abbreviations*: RAP: right atrial pressure; PAP: pulmonary artery pressure; 6MWD: 6-minute walk distance; PEA: pulmonary endarterectomy; BPA: balloon pulmonary angioplasty; sGC: soluble guanylate cyclase; ERA: endothelin-receptor antagonists; PDE5-i: phosphodiesterase type-5 inhibitors

## Discussion

The present study presented a monocentric cohort of patients with CTEPH with long-term follow-up and demonstrated that patients with CTEPH rarely died of right heart failure, even if hemodynamically severe at diagnosis in the modern management era, where almost all patients have received interventional treatments with PEA or BPA and medical therapy. However, patients with CTEPH frequently had comorbid cancer, which might be a strong prognostic factor. In patients with CTEPH, careful screening and lifelong follow-up may be required for early diagnosis of cancer.

In the past, when no specific treatments of CTEPH were available, prognosis of CTEPH with a mean pulmonary artery pressure > 30 mmHg was poor, with a 5-year survival rate of 40% [4]. An international prospective registry by Delcroix et al. from 27 European centers followed up 679 patients with CTEPH diagnosed between 2007 and 2009. At that time, only PEA and off-label use of drugs for treatment of CTEPH were available. In that registry, 404 (59%) patients underwent PEA and non-operated patients (*n* = 275, 41%) had a significantly worse prognosis, with a 3-year survival rate of 70%, whereas that of patients undergoing surgery was 89% [15]. BPA has become available as an additional treatment option in patients with non-operable CTEPH. The first case series reported by Feinstein et al. (2001) showed that its efficacy and safety were not satisfactory [16]. With refinements in this technique, several studies, mainly from Japan, have reported the efficacy and safety of BPA [17, 18]. Moreover, with the accumulation of evidence, attempts to treat non-operable CTEPH with BPA have spread to several countries outside Japan [19–21]. Recently, data from the French PH registry have shown that prognosis of non-operated patients with CTEPH diagnosed between 2013 and 2016 after the availability of BPA improved, with a 3-year survival rate of 85.0%. In that study cohort, 80 of 170 non-operated patients (47.1%) underwent BPA, which was independently associated with improved survival [22]. In recent years, monocentric cohorts of CTEPH, where 90% of non-operated patients with CTEPH had been treated with BPA and approved PAH drugs were concurrently used in half of the patients, have shown excellent survival rates of non-operated CTEPH, with a 3-year survival rate of 91.8% [7]. In addition to conventional PEA treatment of operable CTEPH, BPA and approved pulmonary vasodilators have become established treatment options of non-operable CTEPH in the current guidelines with a class I recommendation [5]. Almost all patients with CTEPH can be satisfactorily treated with either PEA or BPA at a CTEPH expert center, which would lead to a notable improvement in their prognosis. The present study showed that the most frequent cause of death in patients with CTEPH was not right heart failure, but cancer during follow-up in the recent management era.

With improvements in treatment methods and prognoses, prognostic factors have also changed. In the aforementioned international CTEPH registry of patients diagnosed between 2007 and 2009, non-operable disease, advanced age, severe functional status, higher right atrial pressure, cancer, and dialysis were independently associated with poor prognosis [15]. After the availability of BPA for non-operable CTEPH, a higher 6-MWT distance and receiving BPA were independently associated with survival in the French PH registry [22]. In the present study, only the comorbidities of cancer and dialysis-dependent renal failure were associated with poor survival. A simple comparison between studies may not be appropriate because the patients’ backgrounds and numbers differed. However, severe hemodynamics, severe functional status, and poor exercise capacity were no longer prognostic factors in the recent period where three treatment options were established.

Cancer is associated with CTEPH. In our study, 18% of the patients with CTEPH had comorbid cancer. The International Prospective CTEPH registry showed that 12.7% of 679 patients with CTEPH had a history of cancer at diagnosis [23]. Another European cohort from four large PH centers reported that a history of malignant disease was more frequently observed in patients with CTEPH than in those with PAH (12.2% vs. 4.7%) in a study of 433 patients with CTEPH and 254 patients with PAH [9]. Several epidemiological studies have shown that malignancy is an independent risk factor for thrombosis [9, 15]. Patients with malignancy are at six times higher risk of both initial and recurrent venous thromboembolic events than are healthy individuals [8]. The interaction of tissue factors, activation of the coagulation and fibrinolytic systems, acute phase reaction, inflammation, necrosis, and cytokine production by tumor cells are expected to be involved in the development of venous thrombi in patients with cancer [24, 25]. Chemotherapy, including molecular targeted drugs and other treatments, may also be a risk [26, 27].

Another finding of this study was the timing of diagnosis of cancer and CTEPH. Almost all patients with comorbid cancer were diagnosed within 2 years of or after the CTEPH diagnosis (Fig. 2). The international CTEPH registry of the European PH cohort assessed cancer comorbidities at the time of CTEPH diagnosis [9, 15]. However, almost half of the patients with cancer were diagnosed after CTEPH, and most of them had no signs of malignant tumors at the time of CTEPH diagnosis in this study. Due to the time lapse between the diagnosis of CTEPH and cancer, comorbid cancer was assigned as a time-varying covariate in the Cox proportional hazards model to assess the prognostic factors. However, malignant cancer remained strongly associated with poor survival. As cancer may be diagnosed several years after the diagnosis of CTEPH, careful screening and follow-up may be required for its early diagnosis of cancer. Further studies are required to better understand the association between CTEPH and cancer.

### Limitations

This study has some limitations, the main one being its monocentric and retrospective observational nature. Therefore, the occurrence of missing values for characteristics or hemodynamics was unavoidable, which may have influenced the results of the multivariate regression model. Many of the patients had received cancer treatment at other hospitals. Furthermore, the stage of the disease was unknown in 38.3% of the patients with cancer. Therefore, the sample size was small to compare prognosis based on stage of disease, primary site of cancer, and treatment method. Another limitation was that we did not consider the effects of other associated medical conditions that increase the risk of CTEPH (such as prior splenectomy or infected pacemaker) because of inconsistent information on these conditions in our hospital records until recently.

## Conclusion

In addition to PEA for operable CTEPH, treatment methods for non-operable CTEPH have evolved with the availability of BPA and approved medical drugs in recent decades. Patients with CTEPH rarely die of right heart failure, regardless of severe hemodynamics at diagnosis, in the recent management era, where almost all patients have received interventional treatments with PEA or BPA and medical therapy. However, patients with CTEPH frequently had comorbid cancer, which might be a strong prognostic factor. Furthermore, the patients with active cancer showed worse prognosis, whereas those with cured cancer exhibited better prognosis, similarly as did those without cancer. For achieving better prognosis in patients with CTEPH, careful screening and lifelong follow-up may be required for the early diagnosis of cancer.

## Data Availability

All clinical data were obtained from medical records in Kobe University Hospital. Data supporting the findings of this study are available from the corresponding author upon request.

## Abbreviations

CTEPH: Chronic thromboembolic pulmonary hypertension
PH: Pulmonary hypertension
PEA: Pulmonary endarterectomy
BPA: Balloon pulmonary angioplasty
PAH: Pulmonary arterial hypertension
6-MWT: 6-minute walk test
